# Paternal epigenetic influences on placental health and their impacts on offspring development and disease

**DOI:** 10.3389/fgene.2022.1068408

**Published:** 2022-11-18

**Authors:** Sanat S. Bhadsavle, Michael C. Golding

**Affiliations:** Department of Veterinary Physiology and Pharmacology, School of Veterinary Medicine and Biomedical Sciences, Texas A&M University, College Station, TX, United States

**Keywords:** epigenetic, sperm, placenta, genomic imprinting, intergenerational, paternal effect, developmental programming of adult disease

## Abstract

Our efforts to understand the developmental origins of birth defects and disease have primarily focused on maternal exposures and intrauterine stressors. Recently, research into non-genomic mechanisms of inheritance has led to the recognition that epigenetic factors carried in sperm also significantly impact the health of future generations. However, although researchers have described a range of potential epigenetic signals transmitted through sperm, we have yet to obtain a mechanistic understanding of how these paternally-inherited factors influence offspring development and modify life-long health. In this endeavor, the emerging influence of the paternal epigenetic program on placental development, patterning, and function may help explain how a diverse range of male exposures induce comparable intergenerational effects on offspring health. During pregnancy, the placenta serves as the dynamic interface between mother and fetus, regulating nutrient, oxygen, and waste exchange and coordinating fetal growth and maturation. Studies examining intrauterine maternal stressors routinely describe alterations in placental growth, histological organization, and glycogen content, which correlate with well-described influences on infant health and adult onset of disease. Significantly, the emergence of similar phenotypes in models examining preconception male exposures indicates that paternal stressors transmit an epigenetic memory to their offspring that also negatively impacts placental function. Like maternal models, paternally programmed placental dysfunction exerts life-long consequences on offspring health, particularly metabolic function. Here, focusing primarily on rodent models, we review the literature and discuss the influences of preconception male health and exposure history on placental growth and patterning. We emphasize the emergence of common placental phenotypes shared between models examining preconception male and intrauterine stressors but note that the direction of change frequently differs between maternal and paternal exposures. We posit that alterations in placental growth, histological organization, and glycogen content broadly serve as reliable markers of altered paternal developmental programming, predicting the emergence of structural and metabolic defects in the offspring. Finally, we suggest the existence of an unrecognized developmental axis between the male germline and the extraembryonic lineages that may have evolved to enhance fetal adaptation.

## Introduction

**FIGURE 1:**
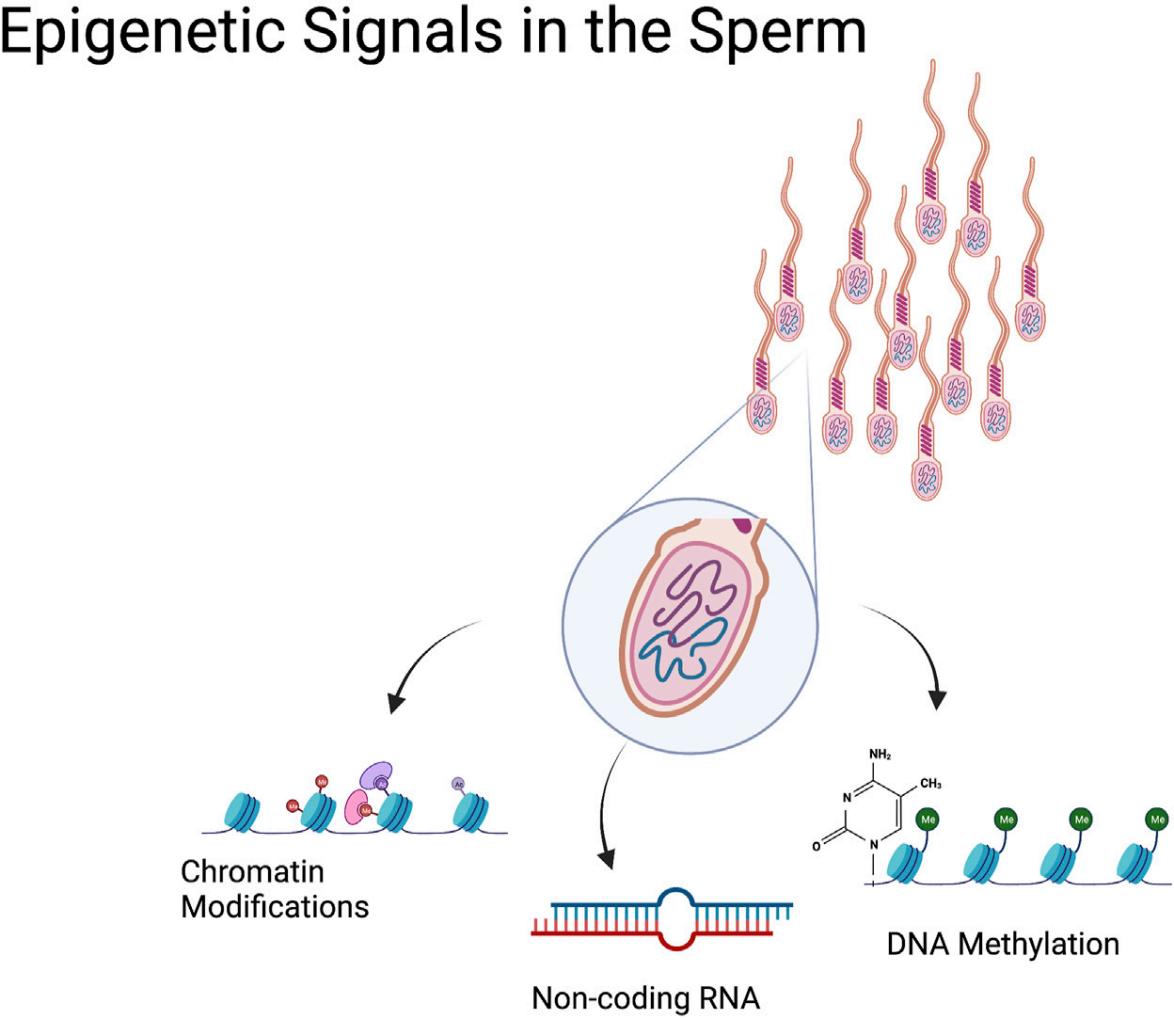
Epigenetic Signals in the Sperm. In addition to the paternal genome, sperm cells transmit epigenetic information from the sire to the offspring. These ‘epigenetic signals’ include a variety of sperm histone posttranslational modifications that are retained during spermatogenesis and may transmit to the developing embryo. Sperm DNA methylation is another epigenetic modification carried by the sperm that has the potential to influence embryonic transcription. Sperm also carry a wide range of noncoding RNAs (miRNA, tRFs, piRNA) that influence zygotic transcription. These epigenetic signals enable the transmission of nongenomic information to the offspring.

Sperm are principally known for carrying DNA, specialized cells that deliver one-half of the genome required to give rise to healthy offspring. However, we now know these cells carry much more than just a haploid set of chromosomes. During spermatogenesis, sperm cells undergo widespread transcriptional and structural changes as they differentiate (Larose et al., 2019). During this process, changes in DNA methylation and posttranslational histone modifications, followed by the sequential replacement of most histones by protamines, yield an incredibly specialized cell type with a remarkably unique epigenome (Le Blévec et al., 2020). Subsequently, during transit through the epididymis, additional epigenetic signals are conferred to sperm as they mature to become fertilization competent, including alterations in noncoding RNAs and additional changes in posttranslational histone modifications (Yoshida et al., 2018; Bedi et al., 2022a; Conine and Rando, 2022) (Figure 1).

Over the past 10 years, clinical and biomedical studies have demonstrated that epigenetic factors carried in sperm significantly influence the health of future generations (Lane et al., 2014; Fleming et al., 2018). These studies have challenged the exclusive importance of gestational exposures in mediating environmentally-induced disease and provide compelling evidence to help redress the notion that exposure-induced birth defects are solely the woman’s fault. Notably, these studies also demonstrate that some aspects of teratogenesis are programmed; epigenetic changes pass through common progenitors to exert successive tissue-specific effects in an ensuing life stage. However, there is still a foundational lack of knowledge concerning how environmental stressors impact epigenetic processes controlling sperm production, and, as yet, the mechanisms by which these inherited epimutations persist to influence offspring health remain almost entirely undefined.

The most plausible track in this endeavor is determining the influence of paternal exposures on the development and function of the placenta. In this review, we seek to understand how changes in sperm-borne epigenetic signals broadly influence offspring health by focusing on the impacts on placental biology. We predominantly focus our review on mouse models, for which a growing body of literature is available. Finally, we will endeavor to explain how altered epigenetic programming in sperm influences embryogenesis and placentation. In doing so, we aim bridge the gap between paternal exposures and pediatric disease and identify potential markers of altered developmental programming common between divergent models examining preconception paternal exposures.

## Preconception paternal stressors and placental function

The placenta is the dynamic interface between mother and fetus that regulates nutrient, oxygen, and waste exchange, coordinates fetal growth, metabolism, and maturation, and determines gestation length. Consequently, factors influencing placental development and function are not only crucial in determining successful pregnancy outcomes; they set the stage for multiple aspects of lifetime health (Burton et al., 2016). Although rarely considered when assessing child-health outcomes, paternally-inherited epigenetic factors have been long-known to play critical roles in controlling the development and differentiation of extraembryonic tissues across multiple mammalian species (McGrath and Solter, 1984; Surani et al., 1984; Wang et al., 2013). Accordingly, several studies examining the intergenerational impacts of paternal stressors report alterations in placental growth within the next generation (Table 1). Below, we review these data and discuss potential mechanistic pathways linking paternal exposures to placental dysfunction while also infusing some caution into the interpretation of these changes.

**TABLE 1:** Studies describing the impacts of preconception paternal exposures on offspring placentation.

Reference	Paternal Exposure Paradigm	Species	Placenta Imprinted Gene(s)	Gross Placental Phenotype	Histological Features
Fornes et al. (2022)	Diabetes	Albino Wistar Rats	Imprinted genes not reported	No significant changes seen	N/A
Jazwiec et al. (2022)	Paternal obesity	C57BL/6J Mice	*Igf2* increased	No significant changes seen	Placental Hypoxia, increased angiogenesis with loss of integrity in vessels
Thomas et al. (2022)	Alcohol (3%, 6%, 10%)	C57BL/6J Mice	*Ascl2, Cdkn1c, H19, Slc22a18, Peg3* dysregulated	Higher Placental Weights for 3% and 6%	Increase in labyrinth and alterations in vascular space
Gao et al. (2021)	Microcystin-LR	Mice	Imprinted genes not reported	No significant changes	Decreased proliferation of labyrinth cells.
					Impaired vasculature in MC-LR exposed placentae
Rokade et al. (2021)	Inflammation	C57BL/6J Mice	No imprinted genes reported	Changes in placental weights	N/A
Thomas et al. (2021)	Alcohol	C57BL/6J: CD1 Hybrid	*Cdkn1c, H19* decreased in females.	Increased placental weight in male C57 offspring, decrease in male CD1 hybrids.	Decrease in junctional zone area and increase in labyrinth in females
			No change in males	Decreased placental diameter in male CD1 hybrids.	
Cissé et al. (2020)	Paternal Stress	C57BL/6J Mice	No imprinted genes identified	No significant changes	NA
Denomme et al. (2020)	Advanced age	CF1 Mice	*Slc22a18, Cdkn1c, Kcnq1, Copg2, Klf14, Igf2r, Slc22a3, Meg3* and *H19* increased.	No significant changes	N/A
			*Kcnq1ot1, Mest, Airn, Ins2* decreased		
McPherson and Lane (2020)	Obesity	C57BL/6J Mice	No imprinted genes reported	No significant changes	N/A
Morgan et al. (2020)	Low protein diet	C57BL/6J Mice	*Igf2, Snrpn, Mest* (No changes reported)	Decreased placental weights	Decreased junctional zone area
Innocenzi et al. (2019)	Cannabinoid receptor agonist	CD-1 Mice	*Peg10* and *Plagl1,* altered methylation	Decreased placental weights	Decreased spongiotrophoblast area with corresponding increase in labyrinth
Ding et al. (2018)	Gestational TCDD	C57BL/6J Mice	*Igf2, H19* decreased	No significant changes	N/A
Gerlinskaya et al. (2017)	Immunization	C57BL/6J, BALB/c, ICR mice	No imprinted genes reported	Changes in placental weights across strains	N/A
Mitchell et al. (2017)	High Fat Diet	C57BL/6J Mice	*Peg3, Peg9, Peg10,* decreased	No significant changes	N/A
Watkins et al. (2017)	Low Protein Diet	C57BL/6J Mice	No imprinted genes reported	Decreased placental weights	Increased Area of Junctional Zone
Binder et al. (2015)	Obesity	C57BL/6J Mice	No significant changes in imprinted genes	Decreased placental weights	N/A
Lambrot et al. (2013)	Folic Acid Deficiency	C57BL/6J Mice	No significant changes in imprinted genes	No significant changes	Decreased spongiotrophoblast area with absence of giant cells
Drake et al. (2011)	Glucocorticoids	Wistar rats	*Igf2* decreased in F1	Placental weights decrease in F1 and paternal F2, increase in maternal F2	N/A
			*Cdkn1c, Phlda, H19* increased in F2		

### Preconception paternal stressors and alterations in placental imprinted gene expression: Causal drivers or additional symptoms?

During the mid-1980s, pioneering studies by McGrath and Solter (McGrath and Solter, 1984) and Surani et al. (Surani et al., 1984) demonstrated that the sperm and egg contain information beyond the genetic code and make unequal contributions to offspring development, with the paternal contribution predominantly driving the growth and differentiation of the placenta and yolk sac. From this early work, the field of genomic imprinting was born, which has since revealed that the appropriate dosage and function of a small cohort of monoallelically expressed genes is critical to controlling fetoplacental development (Bartolomei et al., 1991; DeChiara et al., 1991; Giannoukakis et al., 1993; Constância et al., 2002; Lee and Bartolomei, 2013). Moreover, gene loss-of-function studies examining *Ascl2*, *Cdkn1c*, *Grb10*, *Igf2*, *Igf2r*, *Peg1*, *Peg3*, *Peg10*, *Phlda2*, *Rtl1*, and several others, have revealed that imprinted genes play foundational roles in directing placental differentiation and patterning (Bressan et al., 2009; Piedrahita, 2011).

Notably, multiple aspects of paternal health influence the epigenetic regulation of imprinted genes in sperm, which affects offspring fetoplacental growth. For example, Denomme and colleagues report that age-related changes in sperm DNA methylation are associated with altered placental imprinted gene expression and growth (Denomme et al., 2020). Further, recent clinical studies suggest paternal imprints (here, we reference the inheritance of a silenced paternal allele) may be less stable than maternal imprints, and loss of genomic imprinting impacts placental and infant weight (Vincenz et al., 2020). Therefore, given the established role paternally-expressed imprinted genes have in controlling the development and differentiation of extraembryonic tissues, imprinted genes and their epigenetic regulatory mechanisms represent the logical first suspects in our efforts to understand how paternal stressors and environmental exposures impact offspring fetoplacental health.

Although a relatively small number of studies investigating the influence of paternal exposures on offspring health have examined aspects of placental development, a notable number have identified altered imprinted gene expression (Table 1). For example, placentae derived from the offspring of obese males exhibit altered expression of the imprinted genes *Igf2*, *Peg3*, *Peg9*, and *Peg10* (Mitchell et al., 2017; Jazwiec et al., 2022). Males exposed to exogenous glucocorticoids during gestation sire offspring with reduced placental weights, which correlated with altered expression of the imprinted genes *Igf2*, *Cdkn1c*, *Phlda2*, and *Slc22a18* in both the placenta and fetal liver (Drake et al., 2011). Adult males exposed to the toxicant Tetrachlorodibenzo-p-dioxin (TCDD) display reductions in placental weight and altered placental DNA methylation profiles at the *Igf2-H19* imprint control region (Ding et al., 2018). The offspring of adult males exposed to cannabinoids present with disruptions in the histoarchitecture of the placenta, including reductions in the placental junctional zone and increases in the labyrinth layer, which correlated with altered methylation of the paternally expressed imprinted genes *Peg10* and *Plagl1* (Innocenzi et al., 2019). Likewise, our group has identified changes in placental histology induced by chronic preconception paternal alcohol exposure, in which, similar to the offspring of cannabinoid-exposed males, the labyrinth layer increases and junctional zone decreases (Thomas et al., 2021; Thomas et al., 2022). We also identify alterations in placental imprinted gene expression, including changes in *Ascl2*, *Igf2*, *H19*, and *Slc22a18*. In contrast, placentae derived from the offspring of males maintained on a low protein diet exhibit increased size of the placental junctional zone and a decreased labyrinth layer but also display abnormal expression of multiple imprinted genes, including *Cdkn1c*, *Grb10*, *H19*, *Mest*, and *Snrpn* (Watkins et al., 2017; Morgan et al., 2020). Therefore, paternal exposures appear to transmit a stressor to their offspring, frequently resulting in altered placental imprinted gene expression.

However, determining if alterations in imprinted gene expression are phenotypic drivers or additional symptoms remains a challenging question central to determining how the paternally-inherited epigenetic program influences offspring phenotype. Although human studies suggest genomic imprints transmitted through sperm are more labile than those in oocytes (Vincenz et al., 2020), few studies report correlative DNA methylation profiles between exposed sperm and imprint control regions within offspring placentae. To this point, genome-wide studies using a mouse model of paternal folic acid deficiency, which also reported a thinning of the placental junctional zone and increases in the labyrinth layer, identified 300 differentially expressed placental genes, but only two candidates exhibited differential methylation in sperm; none of the candidates were imprinted genes (Lambrot et al., 2013; Radford et al., 2014). Moreover, neither the aforementioned studies examining paternal glucocorticoid exposures (Drake et al., 2011) nor our work examining alcohol-exposed sperm (Chang et al., 2017; Chang et al., 2019a) identified any DNA methylation changes in sperm or alterations in monoallelic imprinted gene expression. Finally, the remaining studies that report common changes between exposed sperm and offspring placentae identified very modest changes of 2–10%, which previous reports suggest are insufficient to disrupt monoallelic gene expression patterns (Mann et al., 2003; Mann et al., 2004; Susiarjo et al., 2013), and did not employ a mouse model capable of confirming parent-of-origin expression patterns. Therefore, the altered placental imprinted gene expression observed in intergenerational models of paternal exposures are likely additional symptoms and unlikely to represent the primary epigenetic memory influencing offspring phenotypes.

In support of this assertion, recent studies examining the offspring of obese males identify altered *Igf2* expression at gestational day 14.5, but these differences disappear by gestational day 18.5 (Jazwiec et al., 2022). Furthermore, most studies report sex-specific changes in placental imprinted gene expression. These sex-specific patterns and transient alterations indicate that altered imprinted gene expression likely arises as part of a cellular response to a paternally-inherited stressor rather than as a primary driver of altered developmental programming. This argument is consistent with studies examining placental defects induced by assisted reproductive techniques, including superovulation and *in vitro* embryo culture, which do not consistently report altered imprinted gene expression or imprint control region DNA methylation profiles, despite invariably observing placentomegaly and junctional zone overgrowth (de Waal et al., 2015; Chen et al., 2017; Vrooman et al., 2022).

### Preconception male exposures and the epigenetic transmission of placental stressors

**FIGURE 2:**
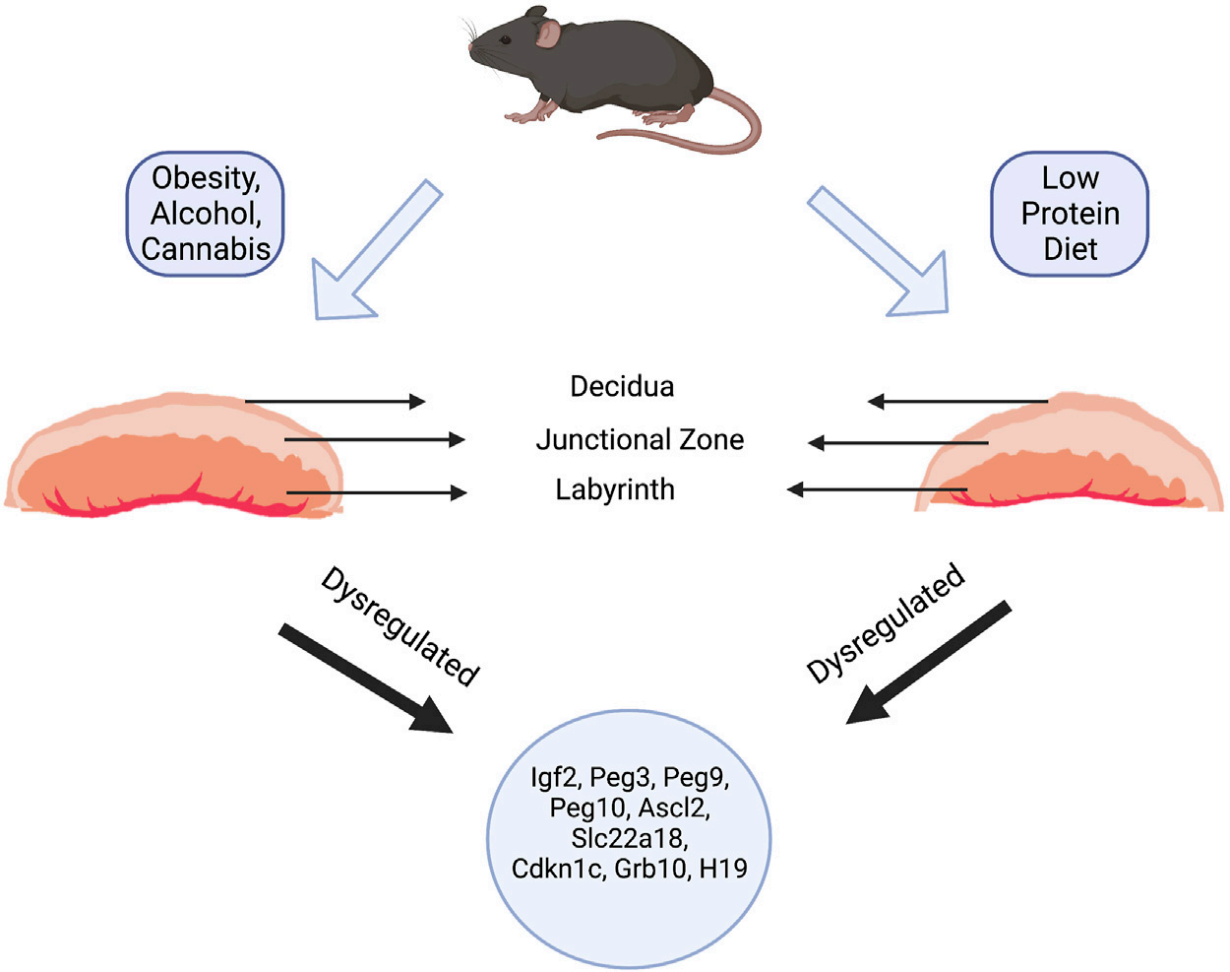
Paternal Exposure and Placental Phenotypes. Paternal exposures induce a wide variety of gross and histological placental phenotypes. For example, paternal alcohol, cannabis, and high-fat diet exposures induce increases in the labyrinth layer (layer responsible for nutrient and gaseous exchange) and decreases in the junctional zone (containing the spongiotrophoblasts and glycogen cells). In contrast, maintaining sires on a low-protein diet induced an increase in the junctional zone and a corresponding decrease in the labyrinth. Regardless of the changes to placental histology, most paternal exposures led to dysregulation of imprinted genes like *Ascl2*, *Igf2*, *H19*, *Slc22a18*, *Cdkn1c*, *Grb10*, *Mest*, and *Snrpn*.

The murine placenta consists of four main histological layers, the chorion, the labyrinth layer, the junctional zone, and the maternal decidua (Figure 2). The functional organization of these layers serves to bring the fetal and maternal blood systems into close contact. Here, the maternal blood supply passes through the spongiotrophoblast cells of the junctional zone *via* a large central sinus. Subsequently, blood becomes distributed into the tortuous, small spaces of the labyrinth, directly bathing the fetal trophoblastic villi. The labyrinth layer, therefore, serves as the primary site of fetomaternal exchange, while the junctional zone functions as the primary endocrine compartment of the placenta, releasing a vast suite of hormones, growth factors, and cytokines that act on both maternal and fetal physiology to regulate pregnancy progression (please see excellent reviews by Rossant and Cross 2001 (Rossant and Cross, 2001) and Woods, et al., 2018 (Woods et al., 2018)).

During times of stress, the placenta allocates priority to the growth and expansion of either the junctional zone or labyrinth, depending on the specific stressor or stage of pregnancy. For example, the processes of superovulation and *in vitro* embryo culture induce an expansion of the junctional zone, accompanied by placentomegaly, reduced placental efficiency, and altered metabolic function in the offspring (Collier et al., 2009; Delle Piane et al., 2010; Bloise et al., 2012; Tan et al., 2016; Chen et al., 2017; Dong et al., 2021; Bai et al., 2022; Vrooman et al., 2022). In contrast, maternal starvation reduces the growth of the junctional zone (at gestational day 16.5), characterized by a prominent reduction in glycogen-producing trophoblast cells (Coan et al., 2010; Sferruzzi-Perri et al., 2011). Decreases and increases in the junctional zone also emerge in loss-of-function studies examining imprinted genes, emphasizing the role these genetic factors have in driving placental histology and adaptation (Tunster et al., 2020).

As briefly mentioned above, multiple studies examining paternal stressors also report changes in placental histoarchitecture, with reallocations primarily occurring between the junctional and labyrinth zones (Watkins et al., 2017; Innocenzi et al., 2019; Morgan et al., 2020; Gao et al., 2021; Thomas et al., 2022). Interestingly, however, these programmed changes often contrast those observed during maternal exposures. For example, in contrast to maternal starvation, which associates with decreased size of the junctional zone (Coan et al., 2010), paternal nutrient restriction programs junctional zone hypertrophy (Watkins et al., 2017). Further, while the offspring of adult males exposed to cannabinoids and alcohol present with reductions in the junctional zone and increases in the labyrinth layer (Innocenzi et al., 2019; Thomas et al., 2022), maternal alcohol exposures promote an expansion of the junctional zone (Gårdebjer et al., 2014). The duality of these responses is intriguing and emphasizes the divergence between paternal and maternal experiences in programming aspects of placental adaptation.

In some reports, paternally programmed alterations in placental histology correlate with altered glycogen content of the junctional zone. For example, paternal toxicant exposures (Ding et al., 2018; Gao et al., 2021), cannabinoid use (Innocenzi et al., 2019), and long-term maintenance on a low protein diet (Watkins et al., 2017) all induce reductions in placental glycogen stores, while chronic preconception paternal alcohol use is associated with increased glycogen levels (Thomas et al., 2021). Reductions in placental glycogen content are also present in mouse models of maternal nutrient restriction (Coan et al., 2010; Sferruzzi-Perri et al., 2011), while studies examining the impacts of assisted reproductive technologies, maternal alcohol use, and gestational glucocorticoid exposures all report increases in placental glycogen (O’Connell et al., 2013; Gårdebjer et al., 2014; Dong et al., 2021). In humans, both decreases and increases in placental glycogen content accompany pregnancy complications that adversely affect fetal development, including intrauterine growth restriction, gestational diabetes, and preeclampsia (Akison et al., 2017). Although we do not yet fully understand the significance of placental glycogen flux, these changes consistently emerge in circumstances where maternal-placental stressors have begun to impact fetal growth (Akison et al., 2017; Tunster et al., 2020).

Because of their glycogen content and location, placental biologists believe spongiotrophoblast cells of the junctional zone serve as a critical energy store, providing additional nutrition to the placenta and/or embryo during specific phases of pregnancy (Tunster et al., 2020). As both maternal and paternal stressors program changes in junctional zone growth and glycogen content, we propose that the placenta’s histological organization and glycogen content offers a dynamic readout of altered paternal epigenetic programming. In support of this hypothesis, we recently reported alterations in placental growth and architecture that varied depending on the dose of alcohol encountered by the father (Thomas et al., 2022). Notably, these dose-dependent changes are non-linear, with low doses inducing placental overgrowth with no histological changes, while higher doses induce growth restriction, which is accompanied by a male-specific reduction in the junctional zone. Combined with other works (Vallaster et al., 2017), these data imply that paternal exposures can program hormetic growth responses, which may bolster offspring toxicant resistance and adaptability to counter adverse environmental conditions.

Although an emerging body of work describes consistent impacts on placental growth and histoarchitecture, the developmental origins of these changes remain obscure. However, a small number of studies report associations between paternal stressors and alterations in early embryonic growth. For example, paternal low-protein and high-fat diets are both associated with delayed progression of embryos through the earliest cleavage events, with the most prolonged delays coinciding with embryonic genome activation around the 2-cell stage (Binder et al., 2012; Sharma et al., 2016). Further, blastocyst-stage embryos derived using sperm from obese males report reductions in the number of cells within the inner cell mass and an expansion of the trophectoderm lineage (Mitchell et al., 2011; Binder et al., 2012). These observations suggest that paternally-inherited epigenetic stressors may impede the earliest phases of embryonic differentiation and lineage specification. Similar to embryos generated using *in vitro* fertilization (Bai et al., 2022), it is plausible that paternal stressors alter the allocation and developmental trajectory of the extraembryonic endoderm, with downstream consequences on placental patterning and function. However, these placental deficiencies may not measurably impact fetal development until late gestation, when the placenta has reached its maximal size and is required to support the near logarithmic increase in late-stage fetal growth (Mu et al., 2008). Notably, deficiencies in late gestation are purported to predominantly impact male offspring, which may help explain the emergence of some sex-specific outcomes across multiple models examining the intergenerational impacts of paternal stressors (Kalisch-Smith et al., 2017).

## Alterations in the sperm-inherited epigenome and altered embryonic development

Several lines of evidence have emerged to help explain how epigenetic changes in sperm may impact embryonic development. However, each of these proposed mechanisms has limitations that complicate our understanding of how paternal exposures influence offspring health and morphogenesis. Below, we will briefly review each epigenetic signal and discuss evidence supporting and limiting the involvement of these mechanistic pathways as drivers of paternal epigenetic inheritance.

### DNA methylation

Of the known epigenetic mechanisms examined to date, DNA methylation is the best characterized across all subdisciplines of developmental programming, including studies examining paternal inheritance. Because early studies contrasting DNA methylation across transposable elements and imprinted genes identified correlative patterns between sperm and embryonic tissues, researchers have long suspected this epigenetic modification participates in the paternal transmission of environmentally-induced phenotypes (Monk et al., 1987; Yoder et al., 1997). Supporting this suspicion, nearly every paternal exposure model or stressor examined to date yields some degree of change in the sperm DNA methylome. For example, high-fat and low-protein diets, exposure to stressful conditions, cold, drugs of abuse, and multiple environmental toxicants modify the DNA methylation profiles of sperm (Anway et al., 2005; Ouko et al., 2009; Knezovich and Ramsay, 2012; Martínez et al., 2014; Radford et al., 2014; Wei et al., 2014; Shea et al., 2015; Chen et al., 2016a; Wu et al., 2016a; Chamorro-Garcia et al., 2017; Le et al., 2017; Ly et al., 2017; Baptissart et al., 2018; Sun et al., 2018; Ben Maamar et al., 2019; Innocenzi et al., 2019; Skinner et al., 2019). Further, several of these studies report consistent alterations between the methylation profiles of exposed sperm and gene regulatory regions driving pathological changes in gene expression in adult animals. Thus, these data suggest that some modified loci in sperm may survive embryonic remodeling, persist into adulthood, and associate with pathological changes in gene expression.

However, although bolstered by the emergence of altered methylation in clinical studies examining the sperm of obese males (Donkin et al., 2016), reported changes in DNA methylation are frequently modest and do not reliably align with pathology-associated gene expression patterns in subsequent generations. For example, studies reporting correlative changes in DNA methylation between sperm and affected tissues in the next generation frequently describe differences ranging from 1% to 5% (Martínez et al., 2014; Wei et al., 2014; Wu et al., 2016a; Sun et al., 2018; Innocenzi et al., 2019). These very subtle differences are unlikely to appreciably impact transcription, and, as discussed previously (Shea et al., 2015), the low frequency of these identified changes in exposed sperm cannot account for the consistent penetrance of offspring phenotypes. Further, most studies examining paternal epigenetic inheritance do not consistently report any direct correlations between changes in sperm DNA methylation and alterations in offspring gene expression or only report the emergence of transcriptional dysregulation in similar genomic regions; sometimes megabases away (Radford et al., 2014; Shea et al., 2015; Terashima et al., 2015; Chen et al., 2016a; de Castro Barbosa et al., 2016; Chamorro-Garcia et al., 2017; Chang et al., 2017; Le et al., 2017; Ly et al., 2017; Sun et al., 2018). Therefore, despite early enthusiasm, there is no compelling evidence that, outside of imprinted genes and select transposable elements, the inheritance of this epigenetic modification through sperm stably influences fetal or adult gene expression in the next generation.

As most DNA methylation is stripped off during syngamy (Smallwood et al., 2011; Smith et al., 2012) and the epigenome is heavily remodeled during histogenesis (Guo et al., 2014), it is unlikely that altered DNA methylation in sperm persists through development to influence transcription in fetal or adult tissues directly. However, emerging evidence indicates that some regions may escape the reprogramming wave during early embryonic development (Hackett et al., 2013; Tang et al., 2015; Zhu et al., 2018; Hao et al., 2021), which could impact gene expression within the developing embryo. Although DNA methylation does have causal roles in the transcriptional regulation of imprinted genes, the suppression of transposable elements, and the process of X-chromosome inactivation, its role in controlling the expression of most protein-coding appears to be responsive rather than causal and is frequently context-specific (Bestor et al., 2015; de Mendoza et al., 2022). As single-cell technologies improve, we may indeed track changes in sperm DNA methylation that persist through the erasure at syngamy and impact the initiation of the earliest transcriptional programs diving development. However, these are likely acute changes altering embryonic transcription, not permanent ones driving pathology in adult tissues.

### Histone posttranslational modifications

Although less explored than DNA methylation, a small number of studies examining alterations in sperm histone posttranslational modifications have also emerged. Despite the replacement of most histones with protamines during spermatogenesis, some genomic loci in sperm retain histones, which carry select posttranslational modifications to the zygote. These nucleosome-enriched regions colocalize with regulatory regions of developmentally crucial genes (Gardiner-Garden et al., 1998; Arpanahi et al., 2009; Hammoud et al., 2009; Brykczynska et al., 2010; Erkek et al., 2013; Royo et al., 2016; Yamaguchi et al., 2018; Yoshida et al., 2018) or gene-poor domains enriched in repetitive elements (Zalenskaya et al., 2000; Carone et al., 2014; Samans et al., 2014; Sillaste et al., 2017), depending on the method of analysis. Similar to studies examining DNA methylation, researchers suspect that a subset of these histone-enriched loci transmits to the early embryo to influence embryonic development.

Whether the environment modulates this form of epigenetic information to heritably influence offspring development is still in the initial stages of investigation. However, research reveals that sperm from males exposed to alcohol, a folic acid-deficient diet, and a high-fat diet all display altered amounts of trimethylated histone H3 lysine 4 (H3K4me3) or dimethylated histone H3 lysine 9 (H3K9me2) (Terashima et al., 2015; Claycombe-Larson et al., 2020; Yoshida et al., 2020; Lismer et al., 2021; Bedi et al., 2022b; Cambiasso et al., 2022; Pepin et al., 2022). Inheritance of these changes may directly impact chromatin accessibility in the developing embryo, impacting the earliest transcriptional programs governing lineage specification and developmental patterning. For example, recent work examining sperm derived from alcohol-exposed males identified a significant increase in global levels of H3K4me3 (Bedi et al., 2022b). This increase in sperm-retained histones may alter chromatin decondensation dynamics during syngamy and delay embryonic genome activation (Binder et al., 2012).

Alternatively, regions of the sperm genome displaying altered chromatin enrichment may persist through the early cleavage stages and directly influence gene expression patterns driving early development. For example, recent studies by Sarah Kimmins’s group have revealed that sperm from folic acid deficient males transmit some H3K4me3-modified loci in preimplantation stage embryos, which are associated with deregulated embryonic gene expression (Lismer et al., 2021). Similarly, sperm from obese males exhibit alterations in H3K4me3 that predominantly map to transcriptionally-active loci of the placental genome; regions controlling inflammation, metabolism, and placental glycogen storage, all of which are transcriptionally dysregulated in this model (Pepin et al., 2022). Notably, there was very little to no conservation between the histone changes identified in sperm and adult offspring liver, arguing against the direct inheritance of these changes as drivers of metabolic syndrome. Furthermore, most regions exhibiting altered H3K4me3 enrichment in sperm isolated from obese, alcohol-exposed, or folic acid deficient males localize to gene enhancer regions controlling embryonic patterning (Lismer et al., 2021; Bedi et al., 2022b; Pepin et al., 2022). Therefore, altered chromatin states may transmit to the embryo and alter embryonic transcription directly.

However, many studies suggest that sperm posttranslational histone modifications and larger aspects of chromatin structure are entirely erased, with H3K4me3 stripped from the paternal genome, and most histone H3.3, which is enriched over gene regulatory regions, is extruded in the second polar body (Du et al., 2017; Flyamer et al., 2017; Gassler et al., 2017; Ke et al., 2017; Kong et al., 2018; van der Weide and de Wit, 2019; Liu et al., 2020). In contrast, other studies report the conservation of multiple histone posttranslational modifications, including H3K4me3, and higher-order chromatin folding between the sperm and early zygote (van der Heijden et al., 2006; Dahl et al., 2016; Wu et al., 2018; Alavattam et al., 2019; Jung et al., 2019; Collombet et al., 2020). As the resolution of chromatin mapping techniques continues to improve, researchers will determine how many regions of the sperm genome escape reprogramming in the early embryo and if regions altered by paternal stressors directly transmit to the offspring, impacting early development. However, as with DNA methylation, it is unlikely that any histone modifications persist into adulthood to drive pathophysiological changes in gene expression directly.

An alternative mechanism to direct transmission of histone modifications and DNA methylation could be the interrelationship between the enrichment of these epigenetic signals and the binding of chromatin accessibility factors either in sperm or immediately after fertilization. Here, increases or decreases in chromatin accessibility may influence larger aspects of embryonic chromatin organization and, therefore, not rely on the direct inheritance of histone-mediated epigenetic marks in sperm.

Although few studies have considered this perspective, some reports describe consistent changes in chromatin accessibility despite inconsistent associations between epigenetic signals. For example, rather than the direct transmission of altered DNA methylation across generations, ancestral exposure to the obesogen tributyltin associates with altered sperm chromatin accessibility at essential metabolic genes dysregulated in adipose cells (Chamorro-Garcia et al., 2017). As another, altered enrichment of H3K4me3 in alcohol-exposed sperm correlates with changes in placental CTCF enrichment and disrupted gene expression patterns at gestational day 14.5 (Bedi et al., 2022b). Similar correlations between altered H3K4me3 and CTCF binding site enrichment exist in sperm isolated from males deficient in folic acid (Lismer et al., 2021). In these scenarios, modified chromatin structure in sperm serves as a bookmark for other factors, persisting after the primary epigenetic signals in sperm are lost. In models of transgenerational epigenetic inheritance, this paradigm may explain the inconsistency of differential DNA methylation and histone enrichment in F0, F1, F2, and F3 sperm, despite conserved pathological phenotypes across generations (Beck et al., 2021). Future studies integrating multiple omics approaches across generations are necessary to determine if these separate epigenetic signals and chromatin accessibility interact in the paternal transmission of growth and disease phenotypes.

### Sperm noncoding RNAs

**FIGURE 3:**
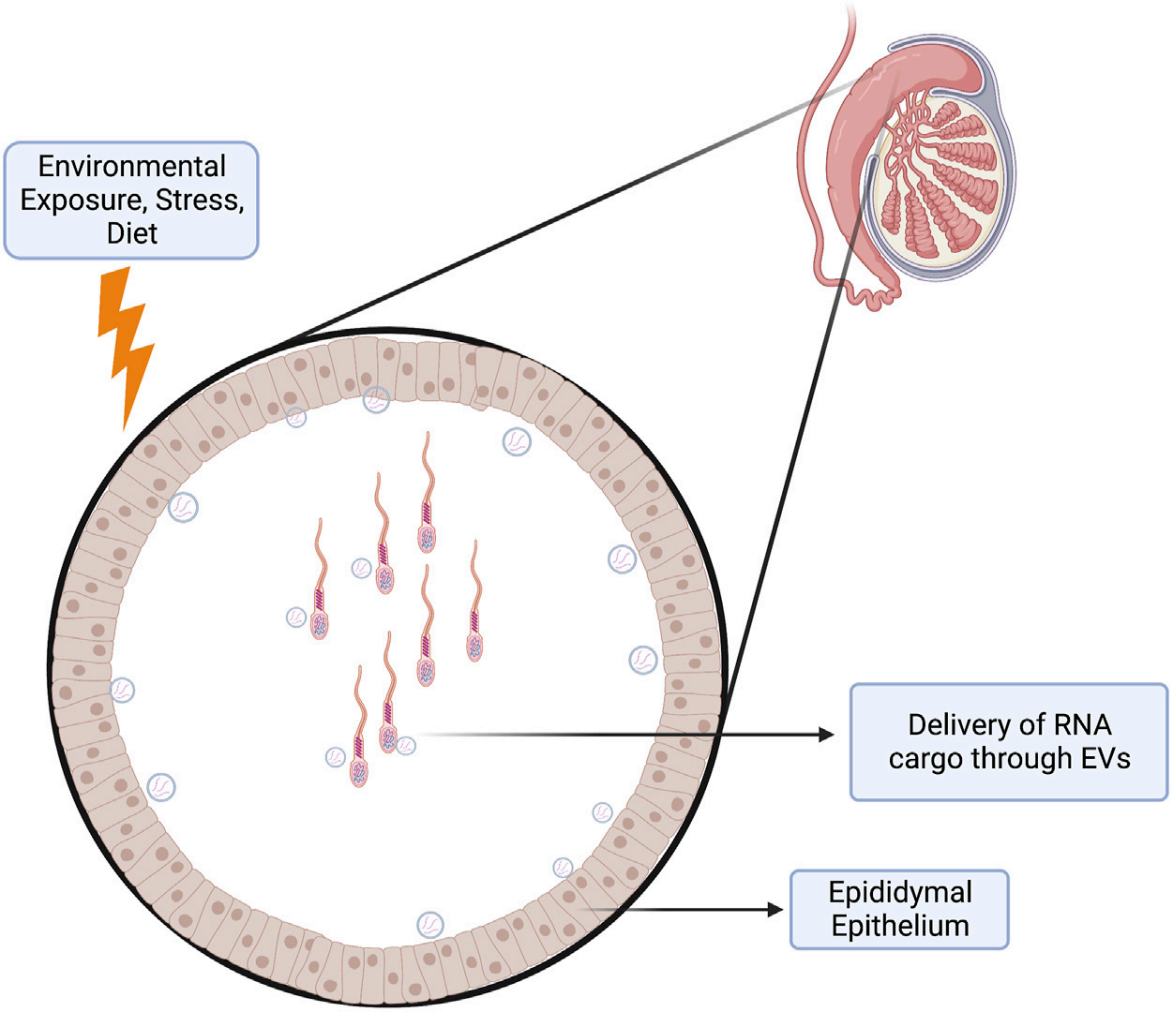
Epididymis as an Environmental Sensor. The epididymal (mainly the caput and corpus) epithelium functions as a sensor of paternal environmental stressors. This epithelium may respond to these stressors by altering its transcriptional program to deliver payloads of molecular cargo through extracellular vesicles (epididymisomes) to the passing spermatozoa. These epididymisomes contain a variety of small RNAs that may deliver a layer of epigenetic information to the maturing spermatozoa, which can alter gene programming events in the early embryo.

**FIGURE 4:**
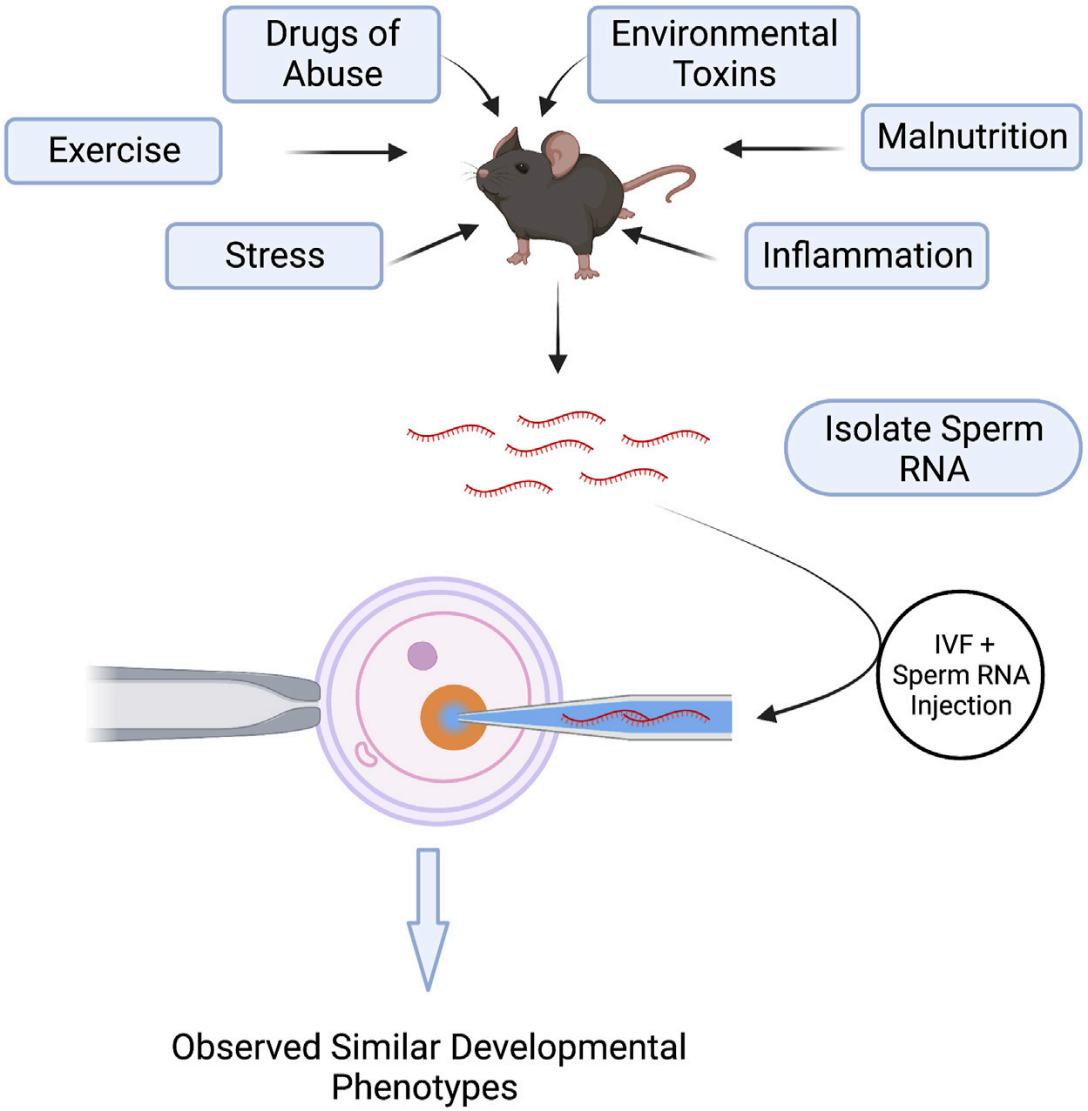
Injection of Sperm noncoding RNAs Recapitulate Environmentally-Induced, Paternally-Inherited Phenotypes in Offspring. Environmental exposures and paternal stressors alter the repertoire of noncoding RNAs carried in sperm. Isolation of these noncoding RNAs from exposed sperm and injection into naïve, *in vitro*-produced embryos induces similar growth and metabolic phenotypes in the offspring as those emerging from *in vivo*-derived embryos. These experiments demonstrate a causal role of sperm noncoding RNAs in the paternal transmission of environmentally-induced phenotypes.

Perhaps the most exciting discovery emerging from studies examining paternal epigenetic inheritance has been the unanticipated influence of sperm noncoding RNAs (ncRNAs) on offspring phenotype (Chen et al., 2016b; Sharma, 2019). Multiple stressors, including exercise, drug abuse, environmental toxicants, inflammation, malnutrition, obesity, and stress, alter the repertoire of sperm-inherited ncRNAs, which correlate with alterations in offspring phenotypes (Conine and Rando, 2022). Many of these ncRNAs originate from extracellular vesicles called epididymisomes, secreted by the luminal epithelium of the epididymis, the portion of the male reproductive tract directing sperm maturation (Zhou et al., 2018) (Figure 3). During epididymal transit, these vesicles fuse with and transmit their ncRNA cargos to maturing sperm (Belleannée et al., 2013; Nixon et al., 2015; Reilly et al., 2016; Sharma et al., 2016; Sharma et al., 2018). Researchers hypothesize that these ncRNAs serve as signaling molecules that modulate genetic pathways driving growth, metabolic, or other adaptive processes in the embryo (Grandjean et al., 2015). Significantly, across multiple experimental models, injection of naive zygotes with small RNAs derived from exposed males is sufficient to induce similar, if not identical phenotypic changes in the resulting offspring (Gapp et al., 2014; Grandjean et al., 2015; Rodgers et al., 2015; Chen et al., 2016a; Conine et al., 2018; Gapp et al., 2018; Sarker et al., 2019; Zhang et al., 2021a; Raad et al., 2021) (Figure 4). Therefore, sperm-derived ncRNAs represent a viable means by which epigenetic information transmits to the embryo to alter physiological function.

Although the mechanisms by which sperm-inherited ncRNAs alter embryonic development remain poorly described, one fascinating theme emerging from studies examining the impact of sperm ncRNAs on embryonic gene expression is an interaction with transposable elements. During preimplantation development, embryos transcribe multiple transposable element families in stage-specific patterns (Vitullo et al., 2012; Fadloun et al., 2013; Liu et al., 2020; Lu et al., 2020; Modzelewski et al., 2021). These transposable elements participate in diverse biological processes, including driving the expression of genes controlling embryonic pluripotency, serving as alternative promoters enabling the generation of novel splice variants, modulating chromatin accessibility to influence the timing of embryonic genome activation, and serving as stage-specific gene regulatory elements (Faulkner et al., 2009; Macfarlan et al., 2012; Elsässer et al., 2015; Wu et al., 2016b; De Iaco et al., 2017; Hendrickson et al., 2017; Jachowicz et al., 2017; Liu et al., 2020; Lu et al., 2020). Importantly, multiple lines of evidence across diverse mammalian species indicate that proper transcriptional control of transposable elements is essential for embryonic development and that manipulating their expression or sequence impacts fundamental aspects of embryo physiology (Beraldi et al., 2006; Jachowicz et al., 2017; Modzelewski et al., 2021). Therefore, sperm ncRNA interactions with transposable element biology may influence multiple facets of embryonic development. This influence may be especially significant for placental development and function, where transposable elements serve as core regulatory elements for this tissue (Rawn and Cross, 2008; Chuong et al., 2013).

Sperm contains a vast repertoire of ncRNAs interacting with transposable elements, including Piwi-interacting RNA (piRNAs), tRNA fragments (tRFs), and microRNAs (miRNAs). PiRNAs are germline-derived small RNAs that direct the transcriptional and posttranscriptional silencing of transposable elements in the male germline (Siomi et al., 2011). Fragments derived from cleaved tRNAs either directly bind multiple clades of endogenous retroviral elements to block their replication or recruit the RNAi machinery to induce their degradation (Schorn et al., 2017). Finally, multiple oocyte-derived miRNAs and endogenous short interfering RNAs map to transposable elements and constrain their expression (Stein et al., 2015). Therefore, multiple small RNA species present in sperm can interact with transposable elements.

A small number of studies tracking the impacts of sperm ncRNAs on embryonic transcription report effects on transposable elements. For example, paternal exposure to a low-protein diet induces alterations in several ncRNA species but most prominently in 5′ fragments of the Glycine tRNA (tRF-Gly). Injection of these tRFs into naive zygotes upregulated genes proximal to the MERVL transposon (Sharma et al., 2016). Subsequent experiments using embryonic stem cells revealed that these tRNA fragments interact with a U7 small nuclear RNA, modulating the translational control of histone proteins; potentially modifying the timing of embryonic genome activation (Boskovic et al., 2020). As highlighted above, paternal low-protein diets retard preimplantation development (Sharma et al., 2016). Similarly, the injection of ncRNAs derived from normal sperm into embryos generated using somatic cell nuclear transfer reduced global levels of H3K9me3, a critical modification constraining transposable element transcription and overcoming a significant barrier in cloned embryo development (Liu et al., 2022). Therefore, sperm-derived ncRNAs may modify transposable element transcriptional activity and their regulatory effects through chromatin-based mechanisms.

Alternatively, sperm ncRNAs may upregulate gene expression *via* direct interactions with the genome. For example, multiple transcripts mapping to transposable element fragments appear in the sperm of males subjected to traumatic experiences (Gapp et al., 2018). Injection of LINE1-derived small RNAs into embryos upregulates LINE1 element transcription, potentially by forming triple-helical RNA-DNA hybrids (Fadloun et al., 2013). Similarly, tRFs identified in the sperm of obese males map to genomic regions near transposable elements and proximal to many genes dysregulated in 8-cell stage embryos (Chen et al., 2016a). These correlative data suggest sperm ncRNAs may exert their transcriptional control by either directly binding to gene regulatory regions (promoters or enhancers) or *via* their proximity to TEs.

Although the correlations with altered transposable element activity and chromatin accessibility are tantalizing, it remains difficult to reconcile the negligible scale of RNAs carried by a single sperm compared to the vast repositories found in the oocyte and early zygote (Yang et al., 2016). Therefore, determining how the minor contribution of sperm-derived ncRNAs exerts a lasting impact on embryonic development and influences adult physiology remains a challenging and unresolved question. Nonetheless, microinjection of cauda-specific small RNAs into developmentally incompetent zygotes generated using caput epididymis-derived sperm improves embryo survival and restores embryonic gene expression (Conine et al., 2018).

Researchers speculate that chemical modifications, including methylation at multiple bases (m5C, m6A, and m1A), increase RNA stability, extending the half-life of sperm ncRNAs until well after fertilization (Chen et al., 2016b). However, despite the enhanced stability these modifications confer, ncRNAs only exist for discrete periods and must stably manipulate gene regulatory mechanisms to achieve a lasting impact on animal phenotype. As an alternative to transposable element-centered interactions, researchers have identified an influence of tRFs on ribosome biogenesis (Kim et al., 2017). Like transposable elements, ribosomal sequences are repetitive and challenging to map. Notably, paternal exposure to a low-protein diet also decreases ribosomal gene expression, which may also explain why low-protein embryos developed slower than controls (Sharma et al., 2016). Alternatively, ncRNAs also recruit chromatin-binding factors like CTCF, which could modify the embryonic developmental program (Kung et al., 2015). However, experiments examining the transgenerational inheritance of metabolic phenotypes suggest that although sperm RNAs can act as vectors of intergenerational inheritance, they do not mediate stable transgenerational transmission of diet-induced metabolic alterations (Raad et al., 2021). Although fascinating, much work remains to determine how the minuscule amount of RNA carried in sperm impacts offspring embryonic growth and long-term health.

## Future directions: The placenta as a mediator of early life mitohormesis and the paternal inheritance of protective adaptations

Although most models of paternal epigenetic inheritance report adverse health outcomes, some studies have identified positive changes potentially conferring protective adaptations to adverse environmental challenges. For example, repeated paternal exposures to sublethal doses of the hepatotoxin carbon tetrachloride (CCl4), constant low-level systemic inflammation, and nicotine all suppressed the fibrotic response in the next generation, improving the wound healing response (Zeybel et al., 2012; Zhang et al., 2020; Zhang et al., 2021a; Zhang et al., 2021b). In addition, paternal nicotine exposure also enhances offspring xenobiotic responses to toxicants by upregulating hepatic detoxification genes (Vallaster et al., 2017).

The mechanisms by which the memories of these stressors achieve protective germline programming remain poorly described. However, many of these reports share similarities with investigations of stressor-induced germline programming in insects, plants, and worms, which also describe enhanced growth, adaptability, and toxicant resistance in the offspring of organisms exposed to low-dose stressors (Agathokleous et al., 2022). In worms, multiple reports can link transgenerational germline programming to early-life mitochondrial dysfunction and the epigenetic regulation of antioxidant pathways (Kishimoto et al., 2017; Zhang et al., 2021c). Significantly, similar pathways are also present in mammalian systems, and transient, intrauterine episodes of placental oxidative stress induce improvements in hepatic metabolism, priming of antioxidant pathways, and resistance to high-fat diet-induced obesity; a phenomenon broadly termed mitohormesis (Yun and Finkel, 2014; Cox et al., 2018; Dimova et al., 2020). Our data examining low-level paternal alcohol exposures also identify altered transcription of placental mitochondrial genes (Thomas et al., 2021; Thomas et al., 2022), and the male offspring of alcohol-exposed sires exhibit resistance to the effects of a high-fat diet (Chang et al., 2019b). Therefore, hormetic alterations in placental mitochondrial function may represent a mechanistic pathway by which paternal exposures program fetoplacental adaptive responses, which may or may not be compatible with the gestational or postnatal environment. Additionally, changes in oocyte mitochondrial function are also observed in maternal models of obesity, suggesting this pathway may not be unique to the male germline.

As discussed above, many placental changes induced by paternal exposures are sex-specific, with paternal stressors inducing diametrically opposite changes in the directionality of affected gene sets between males and females (Chang et al., 2019a; Cissé et al., 2020). It is also noteworthy that emerging research reveals that male cells contain more mitochondria than females (Cao et al., 2022). Therefore, sex differences in mitochondrial function may help explain the sexual dimorphisms observed across studies examining paternal stressors and why males are more sensitive to specific exposures. However, as we know almost nothing about the dynamics of placental mitochondrial function, we require additional studies to determine the validity of this hypothesis.

## Conclusions and future directions

Ultimately, just as maternal exposures do not occur in isolation, a myopic focus on paternal exposures offers limited insights. Although limited in scope, a small number of studies have emerged examining dual-parental exposures to obesity and stress (McPherson et al., 2015; Ornellas et al., 2015; Cissé et al., 2020). Importantly, these studies reveal that maternal and paternal exposures tend to disproportionately impact one sex and, when combined, that parental sex-specific effect becomes exacerbated (Cissé et al., 2020). Moving forward, additional dose-response studies are necessary to determine if environmental stressor-induced transgenerational hormesis plays a prominent role in mammalian development, as in insects, worms, plants, and microbes (Agathokleous et al., 2022). Further, we need to develop more multiplex exposure models to determine how preconception paternal exposures may interact with maternal stressors to influence offspring growth and disease development. Only by examining the combined experiences of both parents will we truly understand the developmental origins of disease. Finally, we believe that, in these endeavors, the placenta offers the best direct readout of altered developmental programming.
